# A patient perspective on recurrent or prolonged contact with psychiatric inpatient care for affective disorder

**DOI:** 10.1186/s13033-018-0205-3

**Published:** 2018-06-05

**Authors:** Emil Brännström, Jennifer Strand, Peter Sand

**Affiliations:** 1 Department of Psychiatry, Sahlgrenska University Hospital, Gothenburg, Sweden; 2 Department of Psychology, Gothenburg University, Gothenburg, Sweden; 3 Department of Psychiatry and Neurochemistry, Institute of Neuroscience and Physiology, The Sahlgrenska Academy, University of Gothenburg, Sahlgrenska University Hospital, Gothenburg, Sweden

## Abstract

**Background:**

The aim of this qualitative study was to explore why some patients receive recurrent or prolonged psychiatric inpatient care, based on the experiences of the patients themselves.

**Methods:**

The participants were recruited at an outpatient clinic at the department of psychiatry for patients with affective disorders at Sahlgrenska University Hospital, Sweden. Ten patients, nine women and one man, aged 22–61 years, agreed to participate. A semi-structured interview guide was used during the interviews, which were audiotaped, transcribed, and analyzed using interpretative phenomenological analysis.

**Results:**

The four themes that emerged were *Difficulties in affective regulation*, where the informants reported difficulty in managing their emotions, with the possible consequence of admission to inpatient care; *Relational sensitivity*, concerning a sensitivity to relationships with healthcare professionals and a need for a secure therapeutic rapport; *Resignation,* characterized by passivity and depression; and *Ambivalence towards responsibility*, where ambivalence about their responsibility could lead to failure to initiate change.

**Conclusions:**

More options beside inpatient care should be available in cases of an urgent need for help. A stable care structure, good cooperation, and long-term planning based on individual needs are pivotal. In the planning of psychiatric care, consideration must be given to the patient’s relational sensitivity. By encouraging patients to actively seek help, we can counteract their resistance and achieve a more effective contact with psychiatric services.

## Background

Psychiatric care in the Western world has been in an ongoing process of deinstitutionalization since the 1950s [1, 2]. Larger centralized institutions in the mental health sector have been discontinued, and the total number of psychiatric hospitals has been reduced. Instead of moving patients from their local communities to institutions for an extended period, efforts are being made to reorganize care and place it in the patient’s immediate area.

In Sweden, there has been a vision of decentralized psychiatric care since the 1970s, in which patients should, as far as possible, be able to live as integrated members of the community. However, there are questions about whether outpatient care and the municipal support systems are adequate for patients with severe mental illness [3].

No country has organized its psychiatry without the possibility of inpatient care, and in decentralized psychiatry, hospital stays have been shortened but the number of rehospitalizations has increased [4]. Up to 50% of patients in psychiatric inpatient care return within 1 year [5]. Many patients do not feel comfortable in inpatient care, which in the worst case can be seen as a repository for those the open care system cannot take care of [4].

There is a group of patients who spend so much time in inpatient care that it has been considered a new form of institutionalization [6]. Some researchers argue that a structural reinstitutionalization is taking place, in which the abandoned mental hospitals are being replaced by other institutions, with an increasing number of beds in forensic psychiatry wards, prisons, and supportive housing [2]. The high rate of rehospitalization in inpatient care is due in part to lack of care and support, and to the absence of a sense of belonging to society [7]. Systematic reviews have described how a well-developed outpatient and support system can reduce rehospitalizations [8, 9]. However, the fact that rehospitalizations only decreased to a certain extent was interpreted as an indication that some patients with certain conditions will always need hospital care. Alternative crisis resolution or management in a homelike environment with access to mobile emergency teams can be more cost-effective, rewarding, and popular alternatives to inpatient care for many patients in crisis [10–13]. However, it is believed that these efforts should be seen as a supplement to inpatient care, as some patients do need hospital care when their condition is more severe.

### Risk factors

There seems to be a group of patients who are treated more extensively in inpatient care than would be justified on the basis of the psychiatric clinical picture. As this may indicate an inadequate health care system causing patient suffering, extensive research has been conducted over the last 30 years on risk factors for rehospitalization [5]. In a review investigating causes of rehospitalization, clinical risk factors such as schizophrenia, personality disorder, and addiction were found, but also social factors such as education and network, and care-related factors such as accessibility and continuity [6]. One study found that lack of support or negative emotions from the patient’s family and social network was a key factor behind rehospitalization [14]. In another study the role of the care system was investigated, and poorly planned discharges and lack of follow-up were identified as risk factors [9]. A third study examined what distinguished patients who were recurrently treated in inpatient care and found risk factors such as chronic illness states, addiction, lack of social network, and social isolation [15]. In a follow-up study, the severity of the illness was found to be the underlying risk factor that distinguished this patient group [16]. One study used a statistical model that would better reflect the recurrent progress of mental illness; the study found that an increased risk of rehospitalization was associated with the diagnosis of schizophrenia and personality disorder, addiction, low level of education, living alone or in the metropolis, unemployment, and low levels of functioning at discharge [17].

Different studies have highlighted various factors that could lead to recurrent hospitalization, with sometimes contradictory variations in clinical, social, and health-related findings. Despite considerable efforts, research has not succeeded in finding a unique risk factor, or agreed on a single explanatory model [5]. The only consensus regarding risk factors for future rehospitalization appears to be for the factors severity of the illness and previous frequent hospitalizations [5, 18], findings that may be of limited clinical value. Moreover, the patients who are hospitalized frequently have many variables in common with those who are not, and there are few factors that separate the patient groups in studies [16].

### Patients’ perspectives on recurrent or prolonged inpatient care

Webb et al. [16] argued that there are scientific challenges in the search for general risk factors for rehospitalization, as the studies often differ in methodology, definitions, and examined care systems. The failure of previous research to find obvious reasons for recurrent or prolonged inpatient care is made worse by the lack of qualitative research and studies of the patient’s perspective on the problem [6]. Psychiatry differs from other areas of healthcare, since the cause of psychiatric illnesses is more contextual and individual, and clear quantifiable and causal relationships are rarely found. The fact that different studies have found different causes of prolonged inpatient care suggests that this is a complex problem that depends on the context. When dealing with contextual issues, qualitative research is valuable for gaining understanding [19].

Smith [18] investigated how the perceived problems of a group of patients contributed to their rehospitalization into inpatient care. Patients described that inadequate support or conflicts within the family or social network are central causes, but also unsafe housing and relapse in addiction. However, the main risk factors suggested by the patients as obstacles to the care they needed were healthcare issues: they lacked a point of contact in the healthcare services and reported inadequate response, accessibility, and continuity. Mgutshini [5] compared patients’ perspectives on rehospitalization in inpatient care with those of healthcare professionals, and reported that many patients emphasized situational factors such as isolation, exclusion, and inadequate support from the environment. In contrast, the healthcare professionals focused largely on medical factors, such as lack of compliance with treatment, diagnosis, or addiction. Patients also expressed contextual understanding and that social stressors often triggered crises. The study indicates that there are no clear reasons for the problem of rehospitalization in inpatient care, but shows the importance of understanding the patient’s perspective and context.

### Aim

Existing research shows ambiguous results concerning which patients are at risk of excessive inpatient care and for what reasons. Recurrent or prolonged hospitalizations are rarely motivated as the best treatment for the psychiatric illness, suggesting a deficient care system that causes suffering and impaired quality of life for the patients; furthermore, these hospitalizations incur high costs and reduce the number of inpatient beds available for other patients who are in need of inpatient care. More studies based on the patients’ own perspectives could enhance our understanding of the problem and improve the way healthcare meets these patients’ needs. Consequently, the aim of the study was to explore why some patients receive recurrent or prolonged psychiatric inpatient care, based on the experiences of the patients themselves.

## Methods

### Participants

The participants were recruited at an outpatient clinic at the department of psychiatry for patients with affective disorders at Sahlgrenska University Hospital, Sweden. Ten patients, nine women and one man, aged 22–61 years, participated.

Seven of the participants were in the care of the personality syndrome team, including patients with personality disorders and the remaining three were in the care of the anxiety and depression team. Patients includes in the study were diagnosed with either depression, anxiety or personality disorder. Patients with psychosis or addiction do not attend the outpatient clinic. All participants were assessed as in need of care between October 2015 and October 2016, on the basis of the number of visits to the psychiatric emergency department, the number of admissions to inpatient care and the number of care days. The number of visits to the psychiatric emergency department for the participants varied between 0 and 14 (median = 4). The number of inpatient admissions varied between 2 and 12 (median = 5). The number of care days ranged from 25 to 155 (median = 85.5).

### Data collection

A semi-structured interview guide was used during the interviews. The interview guide was created in the light of previous research, to cover the most relevant areas for the purpose of the study: psychiatric problems, the experience of psychiatric care, the acute crisis, the social situation, and the informant’s thoughts on healthcare. The structure of the standard interview guide was aimed at gaining a picture of the perceived life-world of the informants [20].

### Procedure

The participants were asked to attend the interview at their current clinic, and nine did so. The tenth interview was conducted in the participant’s home. The face-to-face interviews were conducted in January and February 2017 and lasted between 55 and 80 min. In-depth follow-up questions were used to elicit more detailed responses. The interviews were performed according to the principles described by Kvale and Brinkmann [20], with an openness to the perspective of the informants and an active deepening of their responses, in order to obtain a rich description of their experience of the investigated phenomenon.

### Data analysis

The interviews were audiotaped, transcribed, and analyzed using interpretative phenomenological analysis (IPA), which is considered a useful method for clarifying the informant’s perspective [21]. IPA focuses on exploring how people experience and understand important aspects of their lives. The main theoretical basis for IPA is phenomenology, which emphasizes the lived experience of a phenomenon as a core of knowledge of the phenomenon in question. The second theoretical basis for IPA is hermeneutics, which emphasizes the interpretative nature of humans, and posits that experiences arise in a meaningful context that needs to be taken into account. An interpretative phenomenological analysis is conducted with an awareness of these two aspects of a phenomenon, moving between an exploration of the individual’s experience and an analysis of how it is interpreted, both by that individual and by the researcher.

The interviews were analyzed according to methodological guidelines for IPA [21]. The interview transcripts were first read through several times to become familiar with the informant’s perspective, and then analyzed one at a time, Subsequently, descriptive comments, with phenomenological focus, were written in the right margin. In the next step, these experiences were interpreted, and more abstract concepts were written in the left margin. The conceptual comments were collected in a single document and grouped in preliminary themes. A “mind map” was constructed in order to have an overview for patterns between the different themes, allowing a thematic structure to be created for each interview. The analysis then continued according to the guidelines [21] with the next interview, with a systematic focus on its individual nature, so as not to be unduly affected by the analysis of previous interviews. When all the interviews had been analyzed, the individual thematic structures were studied to find overall patterns in the material. The focus was on establishing a thematic structure that, based on all the interviews, conveyed something meaningful about the phenomenon, but was also grounded in individual experiences. Some themes appeared as pivotal, and both the difference between them and the similarity with the underlying subthemes became clearer. A first theme structure was established and the result was then documented. During this writing process, the thematic structure was further clarified. Some themes and subthemes lacked homogeneity, others could be merged, and finally the thematic structure presented below was created.

## Results

In this study, the thematic structure (see Table 1) refers to the central psychological phenomena as the stories of the informants were perceived. In the description of the subthemes, individual examples are then given as illustrations of what can happen when a person with this problem or need meets the psychiatric care providers. The ambition has been to describe something in general in the informants’ stories, and to express the individual experiences within the framework of this overall structure.

**Table 1:** Overview of the thematic structure

Themes	Subthemes
Difficulties in affect regulation	Inadequate access to psychiatric care Limited continuity of care The importance of conversational supportive therapy Passive inpatient care
Relational sensitivity	Unsatisfactory encounters Destructive actions
Resignation	Insufficient active care
Lack of responsibility	Inner resistance and inadequate support Insufficient guidance

The themes describe the psychological problems of the informants, and the subthemes describe the challenges that may arise in the course of psychiatric care

### Difficulties in affect regulation

All informants reported some kind of mental fragility as a more or less central problem in their lives, and as something that frequently triggered more urgent care needs. Most informants described mental crises that they had difficulty handling on their own, with a deteriorating mental state that often led to loss of emotional control and breakdown, which placed them at risk of hospitalization. The underlying problem is best described as difficulties in affective regulation, where the informants reported difficulty in managing their emotions. The four subthemes describe how difficulty in affect regulation and insufficient individual support are likely to lead to hospitalization.

#### Inadequate access to psychiatric care

Many informants experienced a lack of access to psychiatric care as a central problem, expressing frustration that it was difficult to get the help they needed in situations of mental crisis. They described how a difficulty in affective regulation could escalate into a situation that resulted in hospitalization. Many expressed a wish for help in situations causing psychological distress before reaching the stage of needing protection, that is, a level of care between outpatient visits and inpatient care.

*You are taken seriously only when you have taken an overdose, it has to go so far before you get help. Sometimes you want help to avoid sinking as deep as you can go, but that help does not turn up before then, it becomes like a limbo land, where you are all alone and wait for it to explode.*

Several informants expressed a need for other forms of emergency care than inpatient care, and suggested that alternatives such as emergency calls could be of great help.

*One night when you are in bed and have masses of anxiety and have picked up tablets from the pharmacy and feel that, well, there is one more alternative, I can actually call somewhere, or maybe they have a mobile team./…/In a dream scenario, that would have been something.*

#### Limited continuity of care

Many informants described a low degree of continuity and structure in their contacts with the care services. They experienced that repeated changes of psychiatrists made this contact more complicated, and they described how each healthcare contact person made different assessments and plans for them. The informants described being allowed little participation in or understanding of intervention decisions, and a lack of continuity with other parts of their care provision. They also mentioned how this kind of ambiguity contributed to an experience of uncertainty in situations of mental stress, which further complicated the affect regulation and risk of deterioration.

*The majority of my contact with psychiatry, both in outpatient care and inpatient care, have been evaluations based on very short input from my side and very quick decisions from the healthcare provider.*

One informant, unlike most others, described better contact with healthcare and a sense of security as a result of concrete planning. The informant described how the team worked in a coordinated manner and in dialogue with him, went through his different needs; and he described how that involvement made him feel safer in asking for the help he needed and therefore felt he had the power and motivation to work for a change. His experiences illustrate the perceived difference it makes to have a higher degree of continuity and structure in the contact with healthcare.

*Today, it feels like it has become a slightly bigger step forward with the contact here./…/The fact that the occupational therapist organizes job training and the contacts outside of healthcare, that we talk it through, it begins to move forward from that point of view./*—*/Because my occupational therapist will now be going on maternity leave, we have discussed a replacement for her./…/I will get a psychologist/…/as the next step in this treatment between myself and outpatient care.*

#### The importance of supportive therapy

Many informants highlighted supportive therapy as something that helped them manage more challenging emotions on their own. They sometimes described how the therapy put their problems into perspective, but mainly they highlighted the supportive function. The informants felt that the regular therapy helped them to cope with stressful thoughts and feelings that would otherwise escalate, and that they could accommodate and tolerate stressful experiences if they had an appointment for supportive therapy. Moreover, several informants expressed the need for more treatment, both generally and during more difficult periods.

*If I can’t talk to anyone about how I feel, I’ll build it up within me, and then there will be a lot of catastrophizing, there will be a snowball effect, and everything will be chaos. That part has been important, to have someone who listens, and who you feel you are not burdening in the same way, I can say what I want.*

Most informants were pleased with the supportive therapy itself, but many asked for more, longer, or more available treatment, and did not find that the structure with 45 min sessions per week suited their needs. One informant described a flexible solution which had a supportive functioning.

*Since I got home [from the ward] I have seen [my therapist] more often and it has worked well. I have not had to seek refuge in the hospital, and I know I’ll meet [my therapist] again in 3* *days.*

#### Passive inpatient care

One subtheme that was considered to be particularly relevant in this study was the informants’ recurring descriptions of a passive inpatient care that they perceived to be of limited help. Several informants described hospitalizations where neither active treatment nor support had been initiated in preparation for discharge, which seemed to increase the risk of rehospitalization, because of difficulties to regulate affects that led to the previous admission remained.

*There was no transfer process, you just got thrown out in some way, and then it became too scary, and nothing worked and I got anxiety from it, and then I got anxiety from lots of other things, and then it was just too much. I don’t know why it continued in that way, I did not like being admitted, it was not that I longed to go back, but when it became uncontrollable, there was no other place to turn to.*

### Relational sensitivity

Several informants expressed an emotional preoccupation with their relationship with healthcare professionals in addition to the substantial need for care. They described a sensitivity to the relationship and a need for a safe contact. Their relational sensitivity often interacted with difficulties in affect regulation in a complicated way, where difficult emotions partly highlighted a need for support and increased their sensitivity, which made it difficult to establish the safe relationship needed. Two subthemes emerged to describe how sensitivity to the relationship could increase the risk of hospitalization: the first subtheme highlights the difficulties that arose when psychiatric care failed to create the rapport required for a functioning care relationship; the second subtheme reflects how the breakdown of healthcare encounters could escalate into destructive actions.

#### Unsatisfactory encounters

Several informants talked about recurrent situations in contact with psychiatric care, in which the two had difficulty building rapport, and where care was perceived to have failed to adapt to the patient’s relational needs. The informants expressed a frustration about the inadequate meetings and felt misunderstood and not listened to; they described one-sided interactions in which they were not met in a mutual conversation. The more of these types of meetings an informant described, the worse the healthcare was perceived to function.

*Things that may be small, like wanting to get in touch with your psychologist, when it doesn’t work, it adds a little to my heap of things./*—*/There have been so many changes in my contact with psychiatry, which has been difficult in several ways, it hasn’t been difficult just because of the way I feel, but it has also been difficult as a result of the way I have been treated and not taken seriously.*

#### Destructive actions

The pattern of encounters reaching a deadlock could also escalate and lead to more destructive actions. Several informants described situations where they asserted their own perspective and the psychiatric care staff maintained theirs, without succeeding in changing the interaction or responding in a different way to the patient’s point of view. These loaded situations could result in destructive actions, which were often intentional, leading to hospitalization. One informant gave an example of how drastic it can be in more difficult situations, when the healthcare professional does not succeed in creating a mutual discussion: in her frustration she expressed herself with action.

*Then I said, “you can’t discharge me, I’m not feeling well,” but they said, “but we don’t see that there is a need for you to stay any longer.” I got upset and cried, and said, “okay, then I discharge myself,” and left and swallowed a bottle of pills/…/and they helped me to accident & emergency. So it was really tough that they didn’t listen.*

### Resignation

Most of the informants’ stories focused on problematic interactions with care or difficulties in managing their affect regulation. However, three informants had different experiences, where they did not have the same approach to the psychiatric healthcare services. Their stories were characterized by passivity and depression, and resignation appeared to be the fundamental difficulty. These informants expressed no great dissatisfaction with the availability of healthcare or with the contact itself, and they had difficulty expressing what they wanted.

The subtheme describes how this resignation, together with an insufficient active care provision, risks deepening the depression and necessitating inpatient care.

#### Insufficient active care

Unlike many informants, who mainly described an oscillating anxiety, these informants spoke of a more constant depressed mood that was difficult to influence. They experienced a resignation and hopelessness, and unlike the other informants, they rarely described themselves as seeking help. The psychiatric care services were relatively absent in their stories, and did not seem to have actively attempted to reach them in their most depressed state. This risked perpetuating their situation, leading to recurring depressive relapse and hospitalization. One informant who had never been voluntarily hospitalized to inpatient care said that, in her worsened condition, she did not want help and that she had difficulty believing in change.

*I have tried a lot of things, both medications and therapy, and nothing happens so I feel very resigned. I no longer believe that it will be fine, even though I wish for it I don’t have the energy./…/I used to believe that it will be fine, but it feels so hard when it’s not getting better.*

Another informant described a pattern that had persisted for several years, in which in a desperate resignation she took an overdose, hoping to get medical treatment in the psychiatric ward. The medicine she was given seldom made a difference, and her hope gradually changed to resignation that risks leading to new destructiveness. Thus, the psychiatric care service does not seem to have succeeded in reaching the patient more actively and changing this deadlock.

### Ambivalence towards responsibility

All informants reported some problems with taking responsibility for their own difficulties, such as seeking the help they needed, and taking into account their limitations and needs. This difficulty differs from the previously described difficulties because it was about situations that the informants increasingly needed to manage by themselves. Difficulties in taking responsibility could lead to further deterioration. Two subthemes emerged to describe how the insufficient responsibility of the patient could lead to hospitalization. One subtheme illustrates how seeking help is hampered by inner resistance and inadequate support; the other reflects how insufficient guidance makes it harder to overcome ambivalence towards long-term responsibility.

#### Inner resistance and inadequate support

Several informants described how they could deny some of their limitations and difficulties, which could lead to mental setbacks and reduce the likelihood of making a constructive request for help. Many also felt a resistance to seeking help, as they were ashamed of their problems and worried about being rejected. Difficulties in accepting their problems and asking for help, combined with an inadequate response from healthcare professionals, posed obstacles for those at risk of a deteriorating mood; eventually they would seek help in a more acute manner, which could result in inpatient care. Many informants described a more manifest opposition to seeking help, and the limited availability and sensitivity of the response from healthcare services strengthened their resistance, which risked them being pushed to destructive help seeking strategies.

*I don’t want to take a place, it feels like I’m doing it unnecessarily, that I take up a place somebody else could have./*—*/It’s very difficult to seek help, and especially to the emergency department, and I would like someone to say, “it’s fine that you’ve sought help, now we’ll get to grips with this and try to make the best of the situation.”*

#### Insufficient guidance

Several informants described situations where they needed to choose to resist destructive solutions, and where psychiatric care could affect the outcome to a limited extent. The informants seemed ambivalent about their responsibility and the effort required to act in a long-term constructive way, which increased the risk of the destructive processes of mental deterioration that could lead to inpatient care. A recurring narrative of many informants was that they did not get the support they needed in situations they could not master and that a good deal of their energy was consumed in trying to handle this. Where psychiatric care was available it did not seem to have guided them constructively in what they both could and needed to focus their energy on and do on their own. Several informants described situations where psychiatric care services had suggested particular actions, but they found it an effort to overcome their resistance to making the suggested changes.

*I really need to sleep for example, but when I’m in hypomania, the last thing I want to do is sleep, so that’s a bit difficult, to succeed in doing what you’re supposed to do and not giving up, that’s what’s hardest.*

One informant described difficulties in dealing with her concerns and needing a soothing response. She described how, in anxious times, she doubted her ability to take care of herself and acted self-destructively in order to be taken care of in the ward. This she regretted afterwards, when she “thought with my adult mind,” as she phrased it.

*I take a relapse into the role of the patient,/…/in order to make things bloody easy. Not having to take responsibility, not having to think, not having to feel, but just to like slide along, but I don’t want that kind of life.*

Several informants gave examples of how ambivalence is an inner process that is not easily accessible to psychiatric care professionals. One informant spoke of how studying, through the commitment it required, helped her to deal with her problems in a different way.

*Until then, I could have been ill any length of time, but since I began studying, well nowadays I don’t actually want to be in hospital, or rather I don’t have time because I’m going to take a home exam, I have something else.*

## Discussion

### Need for structured support

The difficulties in affect regulation was the central area of need described by the informants, combined with a risk of inadequate support producing a deteriorating mental state and inpatient care. The most explicit wish expressed by the informants was for an increased availability of emergency care when they were in urgent need of help, rather than having to become ill enough to be hospitalized; they wanted alternatives to inpatient care in these situations. This is similar to what was found in previous studies, where patients, based on their perceived needs in mental crisis, requested alternatives to hospital care, with safe environments, access to counseling support, involvement, and prompt accessibility [10]. Other strategies to prevent hospitalization is acute mobile teams, or access to an emergency phone, both described as helpful in previous studies [7, 22]. Research reviews provide an impression that such requests can work well in practice, as different mobile teams and enhanced crisis management in the home environment can serve as effective and appreciated alternatives to hospital care for up to 80% of the emergency cases [11–13]. Thus, it would be an important area of improvement for psychiatric care to provide alternative emergency support with good accessibility, in order to effectively address the needs of the patient group. It could also result in reduced pressure on emergency care and inpatient care, and enable better accessibility for those whose illness state requires inpatient care.

Overall, many informants expressed the need for increased support, in addition to emergency availability, and said that a lack of continuity and structure in their encounters with psychiatric care contributed to their difficulty in managing their mental illness. There has been criticism of a psychiatric system which, because of insufficient continuity, responds to crises with repeated hospitalization, where some researchers argue that more long-term and coordinated support such as case management is more efficient and helpful [7]. Intensive case management has also been shown to reduce the extent of inpatient care [8]. Some patients are considered to have been left stranded in the wake of modern psychiatry, since the former institutions have not been replaced with a sufficiently developed system of community care and support, and the collaboration between psychiatric and community care, employment offices and social insurance organizations is lacking [1, 3]. Some researchers argue that these patients primarily need a coherent psychiatric care chain, where collaborating organizations work in coordination with all areas of need [11].

Psychiatry would probably stand to gain from focusing on the situation for this patient group, with an improved care structure and longer term planning based on individual needs. Increased access to psychotherapy is an initiative that could reduce the reliance on inpatient care, and there could be more active work in the inpatient setting to change the circumstances that contributed to the individual’s hospitalization. An approach similar to case management, in which the different healthcare services cooperate to create a long-term sustainable situation with adequate support and care, based on the needs of the patient, could increase the patient’s quality of life and be cost-effective.

### Need for a safe relationship

Many informants described repeated meetings with healthcare professionals who showed inadequate mutuality, where they felt misunderstood and not listened to, and they expressed the need for safe relationships regardless of access to support activities. Research shows the importance of an adequate treatment alliance to achieve satisfactory treatment outcomes in psychiatric care [23]. In a study of what patients in both inpatient and outpatient settings considered to be good psychiatric care, both groups emphasized the quality of how they were treated as a person, and the importance of feeling understood [24]. Studies of patients for whom treatment efficacy has been low have emphasized the importance of clearly structured care, but also the importance of creating a safe relationship to be able to work therapeutically [25, 26]. To get therapists to work actively to create a safe relationship with this patient group, and enable a constructive therapeutic relationship, is likely to be an important area of improvement for psychiatric care. It is necessary to work both with the support structures that the patient needs, and to establish the safe relationships within which a change is possible.

### Need for active care

In the narratives of three informants, a resignation was prominent, and this subgroup of patients with more depressive disorders probably have somewhat different needs. These patients would benefit from a more active psychiatric care which takes responsibility for initiating change. Efforts aimed at an increased agency and a more active and social everyday life have been shown to lead to better self-esteem and increased quality of life for unemployed persons with severe mental health problems [27]. The commitment to push for a change needs to come from the outside, and it would probably be constructive to focus on breaking their resignation and stimulating their agency.

### Need for guidance

Several informants described a tendency to deny their difficulties and resistance towards seeking help. Research has shown that barriers to treatment are a key question: where patients did not feel well-treated and cared for, they showed a resistance to seeking care when needed, and an increased risk of mental deterioration and forced hospitalization [18]. The informants in this study expressed something similar, and many did not seek help when they needed it because their resistance increased after having previously felt rejected and belittled. Increased availability would make it easier to seek the help they need, but many patients would also benefit from a more active welcome for those seeking help. Similarly, as an increased availability and continuity can be constructive and cost effective, encouragement to seek help could probably contribute to a more successful care experience for this patient group. It would likely reduce the risk of destructive behavior, and patients could be more effective cared for if they sought help at an earlier stage.

Several informants described an ambivalence towards the effort and the responsibility that came with demanding situations. In one of the studies of patients with low treatment efficacy, they were described how they frequently were ambivalent between wanting to be an independent person and a helpless patient [26]. This is similar to what several informants expressed in our study, as they struggled to independently use the techniques they had learned, but also found it difficult to seek help and distanced themselves from the helplessness they could present. It would probably be valuable for patients if psychiatric care could clarify this ambivalence, and guide them in what they need to do independently. Such a change most likely requires a safe relationship and a functioning support structure. Just as important as understanding patients’ immediate need for psychiatric support is to identify what challenges they will eventually need to deal with themselves. Providing patients with the support they need, as well as encouraging their own responsibility and effort where necessary, is likely to increase their ability to become more independent and to live as freely as possible.

## Limitations and future research

This study has some limitations that should be discussed. One was that five potential informants, who had a history of a high degree of inpatient care, were reluctant to participate. It would have been interesting to share these patients’ perspectives on the problem, since their needs were likely to be greater.

Another limitation is the number of participants. Although the aim of qualitative studies is not to generalize the results, it is important to point out that more participants could have contributed both to more nuanced findings and to more in-depth understanding of recurrent psychiatric inpatient care. Since a majority of the participants in this study were women, a larger group of patients had made it possible to focus on possible variations related to gender. It should also be noted that a disproportionate majority of the participants were diagnosed with personality syndrome. A larger group of patients could have offered richer experiences of frequent inpatient care from patients with other types of affective disorders.

It is important to point out that the participants’ main problems were personality syndrome or depression and anxiety, and that their experiences of extensive inpatient care are likely to differ from those of patients with psychotic disorders or addiction. Even if inpatient care can be a necessary and helpful treatment for patients with affective disorders—as our participants shared experiences of—social issues and outpatient treatment seem to be essential for the wellbeing for this patient group. Inpatient care may more often be an important part of the health care system for patients with psychotic disorders or addiction regardless of contextual factors. It would be interesting to study the experiences of recurrent or prolonged inpatient care in these patient groups.

Another limitation of the study is the subjective nature of the analysis, in which the researcher in an interpretative phenomenological analysis inevitably creates one particular meaning from the material, among other possible meanings. One of the authors works as a clinical psychologist, and has psychological knowledge and clinical experience that constitutes an understanding that might influence the analysis. The motivation to improve patient care could potentially be a driving force for the analysis in the ambition of finding meaningful patterns and potential solutions.

This study examined patients’ perspectives on why they received so much inpatient care. It would be interesting to complement the patients’ experiences with those of the healthcare staff, and to investigate how they perceive the problems. Another finding that would be interesting to study further is the value of creating a safe relationship with this patient group. The creation of the clinical relationship is a complex phenomenon, yet only one party’s perspective was investigated in this study. It would be useful to study more closely how the treatment of this patient group appears to the healthcare staff and what challenges and areas of improvement exist for them.

## Conclusions

In order to avoid deteriorating to the stage of needing hospitalization, more options besides inpatient care are needed in cases of an urgent need of help.

In order to create a sustainable situation over time, with adequate support and care, there is a need for a stable care structure, good cooperation, and long-term planning based on individual needs.

In the planning of psychiatric care, consideration must be given to the patient’s relational sensitivity. This is often a prerequisite for breaking a cycle of conflicts and misunderstandings, as well as achieving a safe environment and reciprocal conversation required for constructive encounters with psychiatric care.

By encouraging patients to actively seek help, we can counteract their resistance and achieve a more effective contact with psychiatric services, with less destructive behavior. The guidance may also assist in helping patients to take responsibility for the challenges they are facing.
